# Hybrid PET/MRI insert: B0 field optimization by applying active and passive shimming on PET detector level

**DOI:** 10.1186/2197-7364-1-S1-A15

**Published:** 2014-07-29

**Authors:** Jakob Wehner, Bjoern Weissler, Volkmar Schulz

**Affiliations:** Department of Physics of Molecular Imaging Systems, Institute for Experimental Molecular Imaging, RWTH Aachen University, Aachen, Germany; Philips Research Europe, Aachen, Germany

Combining PET and MRI into a hybrid device is challenging since both systems might influence each other. A typical interference problem of such a combined device is the distortion of the MRI’s B_0_ field distribution due to the material brought inside the MRI’s FOV which is in particular challenging for small-bore PET-systems. High field homogeneity is needed for a good MRI acquisition in general as well as in certain applications. Typically, active shimming using dedicated coils is applied to improve the field homogeneity. However, these techniques are limited especially for localized distortion profiles with higher-order characteristics caused by PET/MRI inserts. As a consequence, we are exploring the potential application of shimming on PET detector level (for the Hyperion-II^D^ PET/MRI insert), meaning that the distortion profile caused by PET modules is compensated using additional magnetic materials (passive shimming) and DC coils (active shimming). To explore the technique, B_0_ field measurements have been performed using a whole-body phantom in combination with the MRI body coil. An FFE sequence was used to measure distortion maps of DC loops and small magnetic objects (capacitors, ferrites). These distortion maps served as input for a software framework which has been written to perform the field optimization. The implementation was verified by measurements and fits were performed to extract characteristic parameters of the tested objects. Finally, the implemented software framework was used to homogenize a measured distortion map produced by a single PET module by superimposing distortion corrections from additional simulated materials. The resulting superimposed distortion map showed a significantly improved B_0_ field map quality (reduced spectral width and improved homogeneity). The simulated susceptibility distribution will be applied on PET module level and tested in experiments. Results and details about this study will be presented at the conference.

